# Phytochemical capacity of *Nitraria retusa* leaves extracts inhibiting growth of melanoma cells and enhancing melanogenesis of B16F10 melanoma

**DOI:** 10.1186/s12906-015-0743-z

**Published:** 2015-09-02

**Authors:** Jihed Boubaker, Imen Mokdad Bzeouich, Nouha Nasr, Hajer Ben Ghozlen, Nadia Mustapha, Kamel Ghedira, Leila Chekir-Ghedira

**Affiliations:** Laboratory of Cellular and Molecular Biology, Faculty of Dental Medicine, University of Monastir, Rue Avicenne, Monastir, 5000 Tunisia; Unity of bioactive natural substances and biotechnology, Faculty of Pharmacy, University of Monastir, Rue Avicenne, Monastir, 5000 Tunisia; Higher Institute of Medical Technologies of Tunis, Tunis El Manar University, Tunis El Manar, 2092 Tunisia

**Keywords:** Bioactive compounds, B16F10, Apoptosis, Melanogenesis, Tyrosinase, Flavonoids, Polyphenols

## Abstract

**Background:**

Here, phytochemical profile of *Nitraria retusa (N. Retusa*) leaf extracts was identified and their ability to induce apoptosis and inhibiting growth of melanoma cells and enhancing melanogenesis of B16F10 melanoma was evaluated.

**Methods:**

The Apoptosis was evidenced by investigating DNA fragmentation, and Acridine orange/ethidium bromide staining. Amounts of melanin and tyrosinase were measured spectrophotometrically at 475 nm.

**Results:**

Extracts from *Nitraria retusa* exhibited significant anti-proliferative activity after 48 h of incubation. Our result was confirmed by ladder DNA fragmentation profile. All extracts showed also the ability to enhance melanogenesis and tyrosinase activity of B16F10 melanoma cells.

**Conclusion:**

The tested extracts have a significant biological effect which may be due to their bioactive compounds.

## Background

Traditional herbs and plants are widely used for their properties [1], Consequently, research interest in the use of natural products, such as chemical extracts from medicinal plants, herbs, and spices, for the development of alternative chemotherapeutic agents [2] food additives and cosmetic products is considerable [3, 4].

In fact, the World Health Organization (WHO) has emphasized the importance of the traditional indigenous medicines, since a large majority of rural people in the developing countries still use these medicines as the first defence in health care [5].

It was also reported that a high number of new drugs deriving from plant secondary metabolites have been used in the treatment and/or prevention of cancer. For example the antiproliferative activity of flavonoids, polyphenols and sterols against cancers cells was demonstrated by many researchers [6–8].

In this context melanoma is the most aggressive forms of skin cancer, with high metastatic potential and extraordinary resistance to cytotoxic agents [9] and it is resistant to all current modalities of cancer therapy. Drug resistance in melanoma is associated with defects in the apoptotic programme.

In fact some secondary metabolites have been found to suppress growth and proliferation of transformed or malignant cells through induction of programmed cell death or apoptosis [10, 11]. Alesiani et al. [12] demonstrated that melanoma population growth reduction was linked to differentiation processes detected by monitoring some specific markers like melanine synthesis. That’s why we undertook in this study the effect of *Nitraria retusa* leaf extracts on proliferation and melanogenesis of B16F10 melanoma cells.

In our case, we were interested in the leaf extracts of *Nitraria retusa* in order to investigate alternative phytotherapy solutions to current anticancer treatments and preventing cancer development.

## Methods

### Plant material and preparation of extracts

Leaves of *N. retusa* were collected from saline soils in Sahline, a region situated in mid-Tunisia, in December 2006. Their identification was done by Pr. M. Cheieb (Departmentof Botany, Faculty of Sciences, University of Sfax, Tunisia), according to the Flora of Tunisia [13, 14]. A voucher specimen (N.r-12.06) has been kept in our laboratory for future reference. The leaves were hade dried, powdered, and stored in a tightly closed container for further use. Three hundred and fifty grams of powder, from dried leaves, were sequentially extracted in a Soxhlet apparatus (6 h) (AM Glassware, Aberdeen, Scotland, United Kingdom) with hexane, chloroform, ethyl acetate and methanol solvents. We obtained the correspondent extracts for each solvent. Hexane (Hex), chloroform (Chl) and methanol (MeOH) extracts, with different polarities, were concentrated to dryness and the residues were kept at 4 °C. Then, each extract was resuspended in dimethyl sulfoxide solvent (DMSO). Plant materials were screened for the presence of tannins, flavonoids, coumarins and sterols using the methods previously described by Boubaker, et al. [15].

### Cell line and culture

The B16F10 melanoma line was obtained from American Type Culture Collection (ATCC, Manassas, VA) and maintained at 37 °C in a humidified incubator with 5 % CO_2_ at 37 °C, and grown. The cells were culture in RPMI-1640 medium supple- mented with 10 % (v/v) fetal calf serum (FCS, Biowhitaker, Lonza, Belgium), 2 mM glutamine, 1 % NEA (100X), 1 % sodium pyruvate 100 mM (complete RPMI).

### Assay for cytotoxic activity

Cytotoxicity of *Nitraria retusa* extracts against B16F10 cells was estimated by the 3-(4,5-dimethylthiazol-2-yl)-2,5-diphenyltetrazolium bromide (MTT) assay, based on the reduction of the MTT by mitochondrial dehydrogenases in viable cells. The resulting blue formazan product is measured spectrophotometrically [16]. Cells were seeded in a 96-well plate at a concentration of 5 × 10^3^ cells/well and incubated at 37 °C for 24 h in a 5 % CO_2_ enriched atmosphere. The extracts were firstly dissolved in 1 % DMSO, then in the cell growth medium. Cells were incubated again at 37 °C for 48 h with each of the tested extract at concentrations ranging from 10 to 1000 μg/ml. Next, the medium was removed and cells in each well were incubated with 50 μl of MTT solution (5 mg/ml) at 37 °C for 4 h. MTT solution was then discarded and 50 μl of 100 % DMSO were added to dissolve the insoluble formazan crystal. The optical density was measured at 540 nm. Each drug concentration was tested in triplicate.

The cytotoxic effects of the extracts were estimated in terms of cell population growth inhibition percentage and expressed as IC_50_ which is the concentration of extract that reduces the absorbance of the treated cells by 50 % with reference to the control (cells treated with DMSO). The IC_50_ values were graphically obtained from the dose–response curves. We determined IC_50_ values when cytotoxicity resulted more than 50 % at screening concentrations.

### DNA fragmentation analysis

DNA fragmentation was analysed by agarose gel electrophoresis as described by Wang et al., [17], with slight modifications. B16-F10 cells (1.5 10^6^ cells/ml) were exposed to various concentrations of each compounds (IC_50_, IC_50_ and IC_50_ μg/ml of Hex, Chl, EA and MeOH extracts) for 48 h and harvested by centrifugation. Cell pellets were resuspended in 200 μl of lysis buffer (50 mM Tris–HCl, pH 8.0, 10 mM EDTA, 0.5 % N-Lauroyl Sarcosine Sodium Salt) at room temperature for 1 h then centrifuged at 12 000 g for 20 min at 4 °C. The supernatant was incubated overnight at 56 °C with 250 μg/ml proteinase K. Cell lysates were then treated with 2 mg/ml RNase A and incubated at 56 °C for 2 h. DNA was extracted with chloroform/phenol/isoamyl alcohol (24/25/1, v/v/v) and precipitated from the aqueous phase by centrifugation at 14 000 g for 30 min at 0 °C. The DNA solution was transferred to 1.5 % agarose gel and electrophoresis was carried out at 67 V for 3/4 h with TAE (Tris 40 mM, sodium acetate 20 mM, EDTA 1 mM) as the running buffer. DNA in the gel was visualized with ethidium bromide (0.5 μg/ml) under UV light.

### Acridine orange (AO)/ethidium bromide (EB) staining

Cells were cultured in 6-well plate then treated by all concentration of tested extract of *Nitraria retusa* leaves. After 24 h of incubation, cells were collected and washed with PBS followed by staining with 1:1 mixture of AO/EB 5 100 μg/ml stock). Stained nuclei were visualized under a fluorescence microscope [18].

### Determination of melanin content

Melanin release by cells was measured as described in Skandrani et al. [19] with some modifications. Briefly, B16-F10 cells (5 10^5^) were seeded into a 25-cm^2^ culture flask with 5 ml culture medium and incubated at 37 C for 24 h. The cells were then treated with G extract for 48 h. After treatment, melanogenesis (clo- sely related to amont of melanin produced) was estimated from the amount of melanin retained in the cells (intracellular melanin). Adherent cells were detached by incubation in trypsin; 5 10^5^ cells were then placed in tubes and solubilized in 1 ml Triton X-100 (0.1 %). The absorbance (reflecting intracellular melanin content) for each sample was subsequently measured at Scientific, Madison, WI).

### Tyrosinase activity

Tyrosinase enzyme activity was estimated by measuring rate of L-3,4 dihydroxyphenylalanine (L-DOPA) oxidation, as described previ- ously (Skandrani et al. [19] with slight modification. Briefly, cells (5 × 10^5^) were treated with Hex, Chl, EA, and MeOH extracts of *Nitraria retusa* (340, 80, 50 and 100 μg/ml respectively) for 48 h, 10^6^ cells were then resuspended in phosphate buffer (0.1 M; pH 6.8) containing 0.1 % Triton × 100. Lysate was clarified by centrifugation at 17,500 g for 10 min at 4 °C; 400 μl of supernatant was mixed with 400 μl of the substrate L-DOPA (0.15 %), and absorbance was measured spectrophotometrically at 475 nm, every minute for 10 min.

### Statistical analysis

Data were collected and expressed as the mean ± standard deviation of 3 independent experiments and analyzed for statistical significance from control. The data were tested for statistical differences by one-way ANOVA followed by student test using statistica. The criterion for significance was set at *P* < 0.05.

## Results

### Phytochemical study and determination of extract yield, total polyphenol, flavonoid, tannin and sterol contents of *Nitraria retusa* leaf extracts

The highest content of polyphenols was recorded in Chl extract (100 μg/ml Equivalent concentration of gallic acid). The Hex extract showed the presence of an important quantity of sterols equivalent to 31 %. Whereas, MeOH and EA extracts exhibited the highest quantities of flavonoids respectively (146,52 μg/ml and 193.33 μg/ml concentration equivalent quercetin) (Table 1).

**Table 1:** Quantitative phytochemical screening of extracts from *Nitraria retusa* leaves

	Hex	Chl	EA	MeOH
Equivalent concentration of gallic acid (μg/ml)	-	100 ± 3	66*.*25 ± 3	30 ± 4
Equivalent concentration of Quercétine (μg/ml)	-	-	193*.*33 ± 5	146,52 ± 5
Tanins (mg/100 g)	-	-	2*.*26 ± 0*.*5	1,88 ± 3
Sterols (%)	31	10.75	8*.*68 ± 0*.*01	-

Values represent the mean + − SD of three separate experiments

### Cytotoxic activity

**Fig. 1:**
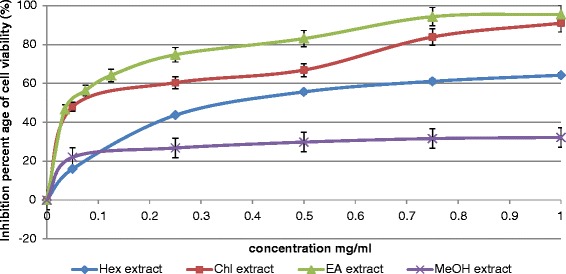
Anti-proliferative effect of *Nitraria retusa* extracts on B16-F10 melanoma cells. Values represent the mean ± SD of three separate experiments

We have examined the effect of different concentrations (from 10 to 1000 μg/ml) of each extract on the B16-F10 cell population growth *in-vitro,* using the MTT assay. The results of this assay were reported in (Fig. 1). EA, Chl, Hex and MeOH extracts inhibited significant the malignant tested cell population growth. (IC_50_ values were 50, 80, 340 and >1000 μg/ ml respectively) (Fig. 1).

### Induction of apoptotic DNA fragmentation by Nitraria retusa extracts on leukemia cells

**Fig. 2:**
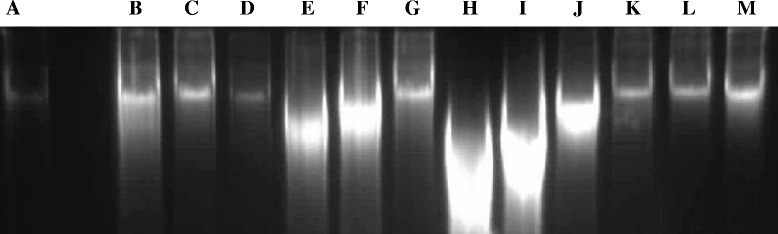
DNA electrophoretic profiles of B16-F10 cells treated with different concentrations of Hex (hexane), Chl (chloroform), EA (ethyl acetate) and MeOH (methanol) extracts during 48 h h. DNA was separated on 1.5 % agarose gel. **a** PC: B16-F10 cell DNA; DNA of cells treated with **b** Hex 340 μg/assay Hex extract, **c** 170 μg/assay Hex extract, **d** 85 μg/assay Hex extract, **e** 80 μg/assay Chl extract, **f** 40 μg/assay Chl extract, **g** 20 μg/assay Chl extract, **h** 50 μg/assay EA extract, **i** 25 μg/assay EA extract, **j** 12.5 μg/assay EA extract, **k** 1000 μg/assay MeOH extract, **l** 500 μg/assay MeOH extract, **m** 250 μg/assay MeOH extract

At exposure with different concentrations of Hex extract (Fig. 2, tracks b, c, d), Chl extract (Fig. 2, tracks e, f, g), EA extract (Fig. 2, tracks h, i, j) and MeOH extract (K,L,M) during 48 h, a fragmented DNA profile was clearly observed in B16-F10 cells treated with Chl and EA extracts, compared to untreated cells which did not provide a ladder DNA profile (Fig. 2, track a).

### Acridine orange (AO)/ethidium bromide (EB) straining

To examine whether the studied extracts induce apoptosis in B16-F10 cells, we assessed cell morphology using the AO and EB straining method.

Morphologically, cells treated with *Nitraria retusa* leaves extracts displayed early apoptotic events including membrane blebbing and chromatin condensation. The apoptotic index reached 40, 38, 32 and 37 % treated with different concentration of hex, Chl, EA and MeOH extracts respectively (Table 2).

**Table 2:** Apoptotic indexes of B16-F10 cells treated with *Nitraria retusa* leaf extracts

Extract	Concentration (μg/ml)	Apoptosis Index (%)
Hexane	85	34 ± 3***
	170	40 ± 2***
	340	29 ± 3**
Chloroform	20	38 ± 2***
	40	32 ± 2***
	80	30 ± 1***
Ethyl Acetate	12.5	23 ± 2**
	25	32 ± 3***
	50	27 ± 1**
Methanol	250	23 ± 3**
	500	36 ± 2***
	1000	37 ± 2***
Negative control	-	1

Values represent the mean + − SD of three separate experiments

The statistical significant of results was evaluated by the Student’s *t*-test. *P < 0.05, **P < 0.01, ***P < 0.001 means significant difference between control and treated sample

### Effect of *Nitraria retusa* leaf extracts on melanin synthesis and tyrosinase activity

To investigate the effect of *Nitraria retusa* leaf extracts on melanin synthesis, B16-F10 melanoma cells were exposed to plant extract at different concentrations for 48 h, and then melanin contents were measured.

In Table 3, results indicate that hexane, chloroforme and ethyl acetate extracts significantly stimulated production of intracellular melanin respectively 29, 18.5 and 18 μg/ml at 340, 80 and 50 μg/ml concentration respectively.

**Table 3:** Effect of extracts from *Nitraria retusa* on melanin content in B16-F10 cells after 48 h incubation

Extracts	Concentration (μg/ml)	Melanin content (μg/ml)
Hexan	340	29***
Chloroform	80	18.5**
Ethyl Acetate	50	18**
Methanol	1000	12
Negative control	-	11

Values represent the mean + − SD of three separate experiments

The statistical significant of results was evaluated by the Student’s *t*-test. *P < 0.05, **P < 0.01, ***P < 0.001 means significant difference between control and treated sample

**Fig. 3:**
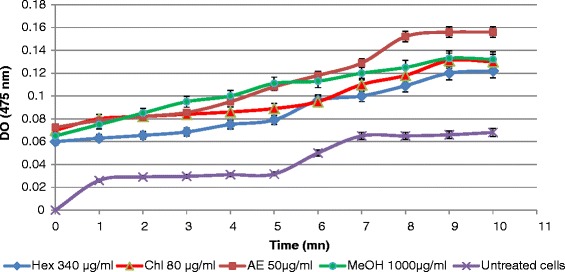
Effect of *Nitraria retusa* extracts on tyrosinase activity in B16-F10 cells after 48 h incubation. Values represent the means ± SD of three separate experiments. The statistical significance of results was evaluated by the Student’s *t*-test. *P < 0.05, **P < 0.01, ***P < 0.001 means significant difference between control and treated sample

Likewise, tyrosinase activity in B16-F10 cells treated with different extracts increases in a time-dependent manner (Fig. 3).

## Discussion

The relationship between concentration of extracts and their antiproliferative effect on B16-F10 cells was investigated by MTT assay. Hex, Chl, EA and MeOH extracts from *Nitraria retusa* exhibited an inhibitory effect on B16-F10 cell proliferation in a dose-dependent manner. Chl and EA extracts exhibited the most important antiproliferative activity. However, no cytotoxic effect was observed on primary culture cells (macrophages, splenocytes) when treated with these extracts at the same tested concentrations (data not shown). Likewise, no mortality was recorded when these extracts were administrated by intra-peritoneal injection in mice even at high tested doses (600 mg/Kg) (data not shown). The cytotoxic activity may be ascribed to the presence of specific components such as polyphenols and flavonoids. As far as our chemical study showed that flavonoids are the main components [11, 20] of our extracts, and as it was previously described for their antiproliferative activity against melanoma cells [21–23], we believe that they are responsible at least in part of our extracts antiproliferative potential. However, many authors [24] have reported that minor components may act synergistically and contribute to anti-proliferative effect of tested extracts.

This finding suggest that the anti-proliferative activity of Chl and EA extracts observed in melanoma cells, B16-F10, could be related to apoptosis. Apoptosis is one of the most prevalent pathways through which chemopreventive/chemotherapeutic agents can inhibit the overall growth of cancer cells [25].

The typical DNA fragmentation pattern which is considered as the hallmark of apoptosis was observed in B16-F10 cells treated with the Chl and EA extracts. As far as the extracts of *N.retusa* tested in the present study were in crude form and probably contained many compounds which may well act synergistically, it is not possible to say which compounds are responsible for the observed effects. However, our data suggest that the biological effects exhibited by this plant, under these experimental conditions, could be related to an overall effect of the tannins, flavonoids, sterols and coumarins compounds in these extracts [11, 20]. These results were in agreement with our previous studies that demonstrated how extracts of *N. Retusa* induce DNA fragmentation which observed after 48 h of incubation with EA extract towards human lymphoblastoid cancer cells, TK6 [20], and with Hex, Chl and MeOH extracts towards human chronic myelogenous erythroleukaemia cells, K562 [11].

The apoptotic potential of *Nitraria retusa* in B16-F10 cells, was also evaluated by cell morphology using the Acridine Orange/ethidium bromide staining. Morphologically, cells treated with *N.retusa* leaf extracts displayed early apoptotic events. The viable cells exhibited a green fluorescence (acridine orange staining) whereas apoptotic cells exhibited an orange-red nuclear fluorescence (ethidium bromide staining) by intercalation of ethidium bromide into DNA damage in apoptotic cells. Indeed, cells in early apoptosis still have their intact membranes therefore have the green core, but not uniformly stained, chromatin condensation occurring in them, cleavage of DNA and/or nuclear fragmentation, these are no longer stuck and its morphology was changed, since cells in late apoptosis show chromatin condensation and orange areas in the nucleus, because in the final stages of the process have lost membrane integrity and ethidium bromide on the predominant acridine orange. In the control group we can observe living cells with nuclei well formed and adhered to the blade [26]. The difference of activity when a large excess of the Hex and EA extracts was added to the assay system, could be explained by the inhibition of the penetration through the cell membrane at high doses of extract components [27].

In order to better understand the mechanism involving in inhibition cell gowth of B16 cells we investigated in our study the effect of our extracts on melanogenesis.

In fact, induction of melanogenesis is considered as a well-known marker of differentiated melanoma cells [28]. It is also reported that the differentiated melanoma is associated with slower cell proliferation [29].

Thus, in the present study, we provide evidence that Hex, Chlo, EA and MeOH extracts exposure effectively stimulate tyrosinase activity and melanogenesis in B16-F10 melanoma cells in a concentration dependent manner. This effect can be attributed to the presence of phenolic components in these extracts. Indeed, diethylstilbestrol, which is a diphenolic component, is able to enhance melanin synthesis in B16 mouse melanoma cells by activation of the cyclic AMP-protein kinase A pathway and upregulation of expression and activity of the melanogenesis-related enzyme tyrosinase − + and microphthalmia-associated transcription factor, a trans-acting factor that regulates the gene transcription of tyrosinase [30]. It is also known that melanin plays an important role in protecting human skin from the harmful effects of UV radiations by absorbing UV sunlight [31]. We can suggest a protective effect of *N. retusa* extracts against skin irritations induced by UV sunlight by enhancing melanogenesis. In fact, as endogenous pigmentation is associated with markedly reduced risk of skin cancer. Agents that enhance skin pigmentation and have the potential to reduce both photodamage and skin cancer incidence took much attention. That’s why, evaluation of topically applied substances that simulate natural pigmentation and substances that stimulate the natural pigmentation process become the target of many studies [19, 32].

In fact, these extracts extracts showed a melanogenesis stimulation activity manner in murine B16-F10 melanoma. However, other plant extracts were reported to inhibit melanogenesis [33], and suppressed melanin synthesis [34].

## Conclusion

*N.retusa* leaf extracts appear to contain compounds with, antiproliferative and apoptotic properties. The three tested extracts induced apoptotic effect by the activation of the extrinsic apoptotic pathway. As apoptosis has become a new therapeutic target in cancer research, it appears reasonable to suggest that *N.retusa* may have potential as an agent of chemotherapeutic and cytostatic activity in murin cancer cells. The tested extracts were also able to induce melanin synthesis and tyrosinase activity and thus the differenciation of B16F10 cells.

## Abbreviations

DNA: Deoxyribonucleic acid
Hex: Hexane
Chl: Chloroform
MeOH: Methanol
DMSO: Dimethyl sulfoxide
AO: Acridine orange
EB: Ethidium bromide
